# Intrinsic activation of the vitamin D antimicrobial pathway by *M*. *leprae* infection is inhibited by type I IFN

**DOI:** 10.1371/journal.pntd.0006815

**Published:** 2018-10-09

**Authors:** Kathryn Zavala, Carter A. Gottlieb, Rosane M. Teles, John S. Adams, Martin Hewison, Robert L. Modlin, Philip T. Liu

**Affiliations:** 1 UCLA/Orthopaedic Hospital, Department of Orthopaedic Surgery, David Geffen School of Medicine at UCLA, Los Angeles, California, United States of America; 2 Division of Dermatology, David Geffen School of Medicine at University of California Los Angeles (UCLA), Los Angeles, California, United States of America; 3 Institute of Metabolism and Systems Research, University of Birmingham, Birmingham, United Kingdom of Great Britain and Northern Ireland; University of Tennessee, UNITED STATES

## Abstract

Following infection, virulent mycobacteria persist and grow within the macrophage, suggesting that the intrinsic activation of an innate antimicrobial response is subverted by the intracellular pathogen. For *Mycobacterium leprae*, the intracellular bacterium that causes leprosy, the addition of exogenous innate or adaptive immune ligands to the infected monocytes/macrophages was required to detect a vitamin D-dependent antimicrobial activity. We investigated whether there is an intrinsic immune response to *M*. *leprae* in macrophages that is inhibited by the pathogen. Upon infection of monocytes with *M*. *leprae*, there was no upregulation of CYP27B1 nor its enzymatic activity converting the inactive prohormone form of vitamin D (25-hydroxyvitamin D) to the bioactive form (1,25α-dihydroxyvitamin D). Given that *M*. *leprae*-induced type I interferon (IFN) inhibited monocyte activation, we blocked the type I IFN receptor (IFNAR), revealing the intrinsic capacity of monocytes to recognize *M*. *leprae* and upregulate CYP27B1. Consistent with these *in vitro* studies, an inverse relationship between expression of CYP27B1 vs. type I IFN downstream gene OAS1 was detected in leprosy patient lesions, leading us to study cytokine-derived macrophages (MΦ) to model cellular responses at the site of disease. Infection of IL-15-derived MΦ, similar to MΦ in lesions from the self-limited form of leprosy, with *M*. *leprae* did not inhibit induction of the vitamin D antimicrobial pathway. In contrast, infection of IL-10-derived MΦ, similar to MΦ in lesions from patients with the progressive form of leprosy, resulted in induction of type I IFN and suppression of the vitamin D directed pathway. Importantly, blockade of the type I IFN response in infected IL-10 MΦ decreased *M*. *leprae* viability. These results indicate that *M*. *leprae* evades the intrinsic capacity of human monocytes/MΦ to activate the vitamin D-mediated antimicrobial pathway via the induction of type I IFN.

## Author summary

Our macrophages are equipped with the ability to detect and kill invading pathogens, and yet, these cells of the innate immune system are still subject to infection by intracellular bacterium. In particular, mycobacterium, the type of intracellular bacteria responsible for diseases such as tuberculosis and leprosy, are very successful at establishing infection within macrophages. By studying *Mycobacterium leprae*, the etiological agent of leprosy, we describe an immune evasion mechanism whereby this bacterial pathogen utilizes our own antiviral immune response against the macrophage. Type I interferons (IFN) are a major part of our immune response to viral infections; however, this response will also suppress our ability to fight opportunistic bacterial infection. During infection of our macrophages, *M*. *leprae* induces an aberrant type I IFN response that subsequently suppresses our macrophage’s ability to activate the vitamin D-mediated antimicrobial pathway, a critical antimicrobial response for containment of mycobacterium. Thus, understanding how these pathogens can evade our immune response will be important for the development of new therapies against these chronic infections.

## Introduction

The ability of macrophages (MΦ) to kill intracellular pathogens is critical to the outcome of infection. Addition of exogenous ligands derived from the pathogen such as a Toll-like receptor 2 ligand (TLR2L) or from human immune cells, such as IFN-γ, provides an extrinsic signal to activate a vitamin D-dependent antimicrobial activity against mycobacteria in infected MΦ [1, 2]. In the absence of these exogenous stimuli, there is evidence for intrinsic antimicrobial mechanisms, although these are not clearly defined nor sustained [3]. In this regard, microbial pathogens have evolved mechanisms to evade these host defense pathways, establishing infection that results in clinical disease. Here we investigated whether in the absence of exogenous triggers such as TLR2L or IFN-γ, there is intrinsic activation of a vitamin D-dependent antimicrobial pathway in infected human MΦ, as well as the presence of bacterial mechanisms of escape, by studying leprosy as a disease model.

Leprosy, caused by the intracellular bacterium *Mycobacterium leprae*, presents as a spectrum of disease in which the clinical manifestation correlates with the immunological state of the patient. At one end is the self-limiting tuberculoid form (T-lep) and the other end is the disseminated lepromatous form (L-lep) [4], thus comparisons of each form of leprosy affords the opportunity to uncover the critical intrinsic immune mechanisms needed to contain the infection. Each pole of the leprosy disease spectrum is accompanied by a distinct immunological profile defined by specific T cell subsets, MΦ populations and cytokine patterns [4]. Gene expression profiling of skin lesions from patients with the different forms of leprosy suggested a correlation between activation of the vitamin D pathway and favorable disease outcomes [5].

For over a century, the potential use of vitamin D as a treatment against pathogenic infections has been investigated [6–11]; however, only in recent years has the mechanisms by which the immune system utilizes vitamin D to mount an antimicrobial response been described. Human monocytes and MΦ can synthesize the active vitamin D hormone 1α,25-dihydroxyvitamin D (1,25D) from the inactive prohormone substrate 25-hydroxyvitamin D (25D) upon stimulation via innate or adaptive immune signals resulting in an antimicrobial response to infection [1, 2, 12]. Conversion of 25D into 1,25D is mediated through the enzymatic actions of 25-hydroxyvitamin D 1α-hydroxylase (CYP27B1). Studies performed *in vitro* with human MΦ have demonstrated that this vitamin D metabolic system plays a role in human MΦ antimicrobial activity against *M*. *tuberculosis* infection as well as in patients with tuberculosis [1, 2, 5, 12, 13].

As the role of vitamin D in the human immune response against *M*. *tuberculosis* infection becomes increasingly established, studies have also emerged linking these mechanistic findings to other human mycobacterial diseases, in particular, leprosy [5, 14–17]. Expression of the vitamin D pathway during disease may be an important facet to disease pathogenesis; therefore, it is important to understand the factors that regulate the expression and function of this antimicrobial pathway. By comparing the gene expression profiles of lesions derived from T-lep vs. L-lep patients, we were previously able to determine that *M*. *leprae* infection of human MΦ resulted in the induction of type I IFN, which directly inhibited activation of the vitamin D-dependent antimicrobial response triggered by exogenous addition of type II IFN (IFN-γ) [17]. Here, we investigated whether the vitamin D antimicrobial pathway is intrinsically activated, in the absence of TLR2 and IFN-γ by *M*. *leprae* infection of MΦ, Furthermore, we evaluated whether the ability of *M*. *leprae* to induce type I IFN blocks the intrinsic activation of the vitamin D pathway, representing an escape mechanism by which the bacterium evades the host response.

## Materials and methods

### Ethics statement

This study was conducted according to the principles expressed in the Declaration of Helsinki, and was approved by the Institutional Review Board of the University of California at Los Angeles. All donors provided written informed consent for the collection of peripheral blood and subsequent analysis.

### Statistics

Experiments comparing two conditions were analyzed using two tailed Student’s t-test. Multiple condition experiments were analyzed using one-way ANOVA with pairwise analysis using Newman-Keuls test. Other tests used are indicated in text. All data used to generate the figures presented are available as S1 Data.

### Immunohistochemistry

Skin biopsy specimens were collected from untreated patients at the Hansen’s Disease Clinic at Los Angeles Country and University of Southern California Medical Center as well as the Leprosy Clinic at the Oswaldo Cruz Foundation in Brazil. The diagnosis and classification of patients were determined based on clinical and histopathological criteria of Ridley and Jopling [4, 18]. Cryosections of skin lesions from T-lep, and L-lep patients were labeled for CYP27b1 (Santa Cruz Biotechnology, Dallas, TX). Sections were incubated with normal horse serum followed by incubation with CYP27b1 antibody or matched isotype control for 60 minutes. Following primary antibody incubation, sections were incubated with biotinylated horse anti-mouse IgG and visualized by ABC Elite system (Vector Laboratories) and AEC Peroxidase Substrate Kit (Vector Laboratories). The section was then counterstained with hematoxylin and mounted with crystal mounting medium (Biomeda). Skin sections were examined using a Leica microscope (Leica 2500). Expression of CYP27b1 in photomicrographs was quantified using ImmunoRatio (http://153.1.200.58:8080/immunoratio/), an automated image analysis application that calculates the percent diaminobenzidine (DAB)-stained nuclear area per total area [17].

### Primary human monocytes

Peripheral blood mononuclear cells (PBMC) were isolated using Ficoll (GE Healthcare, Chicago, IL) density gradient from whole blood obtained from healthy donors with informed consent. Monocytes were enriched using plastic adherence in which PBMCs were cultured for two hours in RPMI 1640 media (Invitrogen, Carlsbad, CA) with 1% FBS (Omega Scientific, Tarzana, CA) followed by washing [2, 12, 14]. Monocytes were cultured in RPMI media with 10% FBS and treated with 100ng/mL Pam_3_CSK_4_ (TLR2L), or *M*. *leprae* (MOI 10, gift from J.L. Krahenbuhl) immediately following adherence. For blocking type I IFN signaling, monocytes or macrophages were treated with 10μg/mL anti-IFNAR antibody (PBL, Piscataway, NJ).

### High-performance liquid chromatography (HPLC)

Following treatments as indicated in the text, monocytes or MΦ were incubated with radiolabeled ^3^[H]-25D_3_ (PerkinElmer, Waltham, MA) substrate for 5 hours in serum-free RPMI media. [^3^H]-metabolites were purified using a C18 column and separated using a Zorbax-sil column (Agilent, Santa Clara, CA). Radioactivity was measured in each sample by scintillation counting. The amount of each metabolite present was quantified from counts per minute plotted against elution profiles of standards for 25D_3_, 1,25D_3_, and 24,25D_3_ [12].

### Quantitative polymerase chain reaction (qPCR)

Total RNA was harvested using TRIzol (Thermo Fisher Scientific, Waltham, MA) and cDNA synthesized using SuperScript III Reverse Transcriptase (Thermo Fisher Scientific). mRNA expression levels for CYP27B1, CYP24A1, VDR, CAMP, and IFNB were assessed using the Taqman system and pre-verified primer and probe sets (Thermo Fisher Scientific). Relative expression was quantified by comparing to the housekeeping gene 18S rRNA and using the ΔΔCt method [12, 14]. OAS1 mRNA levels were assessed using the SYBR Green system (Bio-Rad Laboratories, Irvine, CA) compared to the housekeeping gene 36B4 using the ΔΔCt method. 36B4 primers were previously published [14], and OAS1 primers are as follows: forward 5’-GAG ACC CAA AGG GTT GGA GG-3’, and reverse 5’- GGA TCG TCG GTC TCA TCG TC-3’.

### ELISA

Secreted IFN-β protein in the supernatant were measured using VeriKine Human Interferon-Beta ELISA Kit (PBL Interferon Source) following the manufacturer’s protocols.

### Microarray analysis

The microarray experiment was previously published [17]. Gene expression profiles of mRNAs derived from skin biopsy specimens of 16 leprosy patients (T-lep, n = 10; L-lep, n = 6) were determined using Affymetrix Human U133 Plus 2.0 microarrays and analyzed as previously described. The raw gene expression data analyzed in this study are available online through the Gene Expression Omnibus database (http://www.ncbi.nlm.nih.gov/geo/) accession number GSE17763.

### MΦ differentiation

MΦ were differentiated using our previously described rapid protocols; briefly, monocytes were directly treated with 10^3^ U/mL IL-4 (Peprotech, Rocky Hill, NJ), 10μg/mL IL-10 (R&D Systems, Minneapolis, MN), or 200ng/mL IL-15 (R&D Systems) for 48 hours immediately following plastic adherence [5, 12, 13]. Completion of MΦ differentiation was confirmed histologically. Vitamin D supplementation experiments were carried out *in vitro* by the addition of 25-hydroxyvitamin D_3_ (Enzo Life Sciences, Farmingdale, NY) to the extracellular medium of MΦ cultures.

### *M*. *leprae* infection

MΦ were infected with single cell suspensions of *M*. *leprae* at MOI of 10 in antibiotic free RPMI1640 supplemented with 10% FBS for 16 hours; then washed vigorously to remove extracellular bacterium and cultured in RPMI 1640 supplemented with 10% FBS, penicillin and streptomycin.

### Antimicrobial assay

MΦ were infected with *M*. *leprae* as indicated above. Following washing the infected MΦ were cultured with 10% 25D sufficient human serum. Total RNA and DNA were harvested from *M*. *leprae*-infected macrophages as indicated in the text and cDNA was synthesized from the RNA fraction. Bacterial 16S rRNA and *M*. *leprae* repetitive genomic element DNA (RLEP) were measured using quantitative PCR. Bacterial viability was calculated by comparing 16S rRNA to RLEP DNA as previously published [14].

## Results

**Fig 1:**
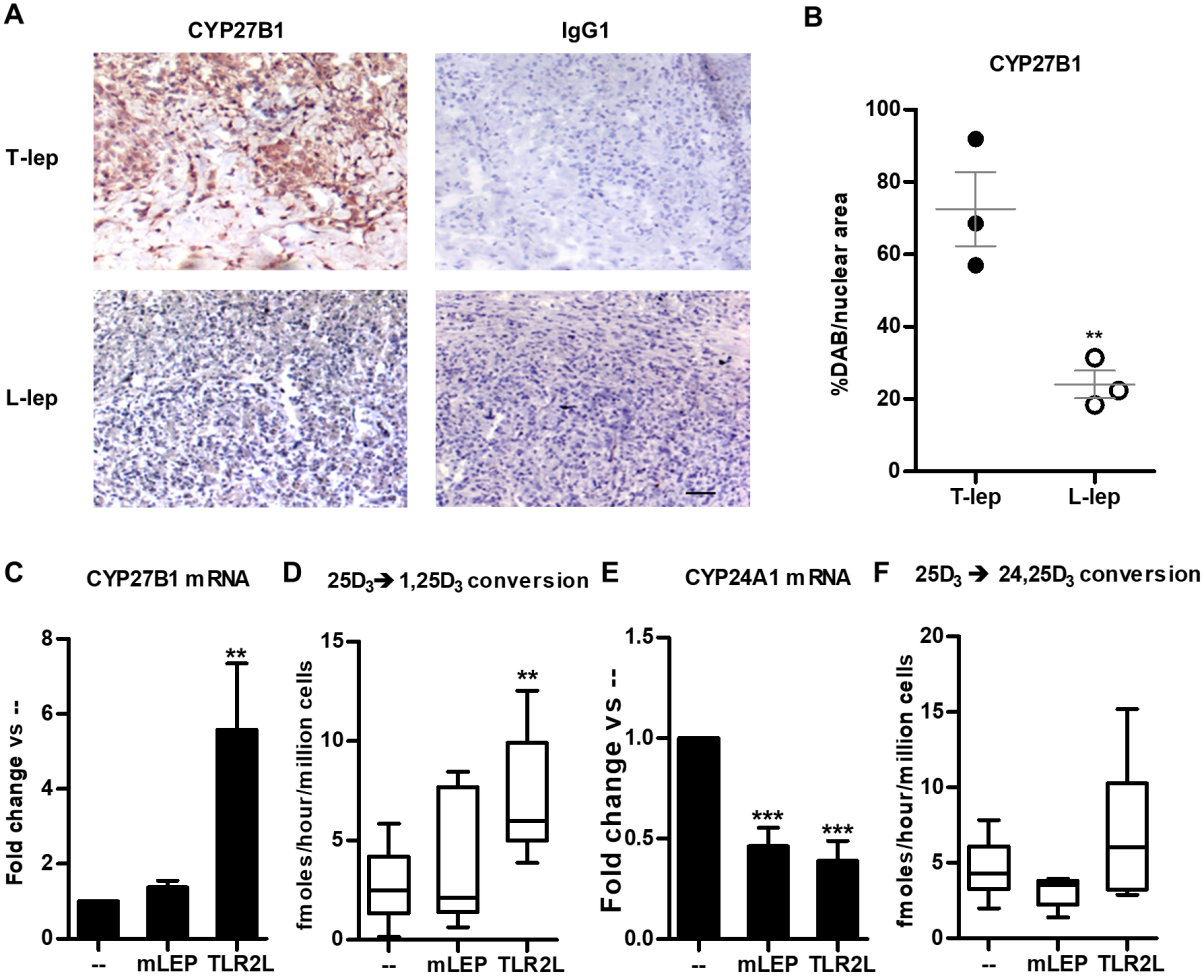
CYP27B1 expression in leprosy and during *M*. *leprae* infection of primary human monocytes. (**A**) One representative labeled section displaying CYP27b1 protein detection in leprosy lesions from one patient, (patient, n = 3), scale bar = 40 μm. Original magnification: x100. (**B**) Automated image analysis of CYP27b1 protein expression. Each dot represents the percentage of CYP27b1–stained area per nuclear area for each individual photomicrograph (n = 3 for each group). Data represent mean ± SEM. Statistical significance was determined using student t-test. Primary human monocytes were treated with *M*. *leprae* at a multiplicity of infection (MOI) of 10 or 100ng/mL Pam_3_CSK_4_ (TLR2L) for 24 hours and (**C**) CYP27B1 and gene expression levels were determined by quantitative PCR, or the cells were incubated with radiolabeled 25D_3_ for 5 hours, and (D) conversion to 1,25D_3_ was measured by high performance liquid chromatography (HPLC). Gene expression of (**E**) CYP24A1 and (**F**) conversion of 25D_3_ into 24,25D_3_ were also measured. Data are shown as mean ± SEM, n >3. Statistical significance was determined using one-way ANOVA. (* p ≤ 0.05, **p ≤ 0.01, ***p ≤ 0.001).

Our previous study indicated that components of the vitamin D-mediated antimicrobial pathway, including CYP27B1 mRNA, were more highly expressed in T-lep lesions as compared to L-lep lesions [5]. Examining the distribution of CYP27B1 protein in leprosy lesions by immunohistochemistry, we observed greater expression of the enzyme throughout the granulomas in the T-lep vs. L-lep form (Fig 1A), correlating with mRNA levels [5]. Quantification of protein expression levels in the leprosy lesions indicated that there were 3-fold higher number of CYP27B1 positive cells in the T-lep vs. L-lep lesions (Fig 1B).

Given that *M*. *leprae* bacilli are more abundant in L-lep lesions, we determined the effect of *M*. *leprae* on CYP27B1 expression and function. Cultures of primary human monocytes were infected overnight with live *M*. *leprae* at a multiplicity of infection (MOI) of 10. Following infection of monocytes, total RNA was isolated, then CYP27B1 mRNA expression was assessed by qPCR. *M*. *leprae* infection did not significantly induce CYP27B1 mRNA expression in monocytes, whereas stimulation with exogenous Toll-like receptor 2/1 ligand (TLR2L) did (Fig 1C). Given that the function of CYP27B1 is to metabolize 25D into 1,25D, we cultured TLR2L activated or *M*. *leprae*-infected monocytes with 25D_3_ for 6 hours, and the levels of 25D_3_, 1,25D_3_ and 24,25D_3_ were measured using high performance liquid chromatography (HPLC). While monocytes treated with the exogenous TLR2L were able to significantly convert 25D_3_ into 1,25D_3_, consistent with previous studies [2], *M*. *leprae*-infected monocytes did not (Fig 1D). Conversely, gene expression of CYP24A1 mRNA, the enzyme that catalyzes 25D into the non-VDR-interacting 25D catabolite, 24,25D, was significantly reduced by *M*. *leprae* infection and TLR2L (Fig 1E). The reduction in CYP24A1 mRNA expression did not result in a detectable change in enzymatic activity (Fig 1F). These results suggest that although *M*. *leprae* expresses a cell wall associated lipoprotein capable of activating TLR2 [19], it does not activate a monocyte intrinsic pathway leading to the induction of CYP27B1 without exogenous TLR2L or IFN-γ.

**Fig 2:**
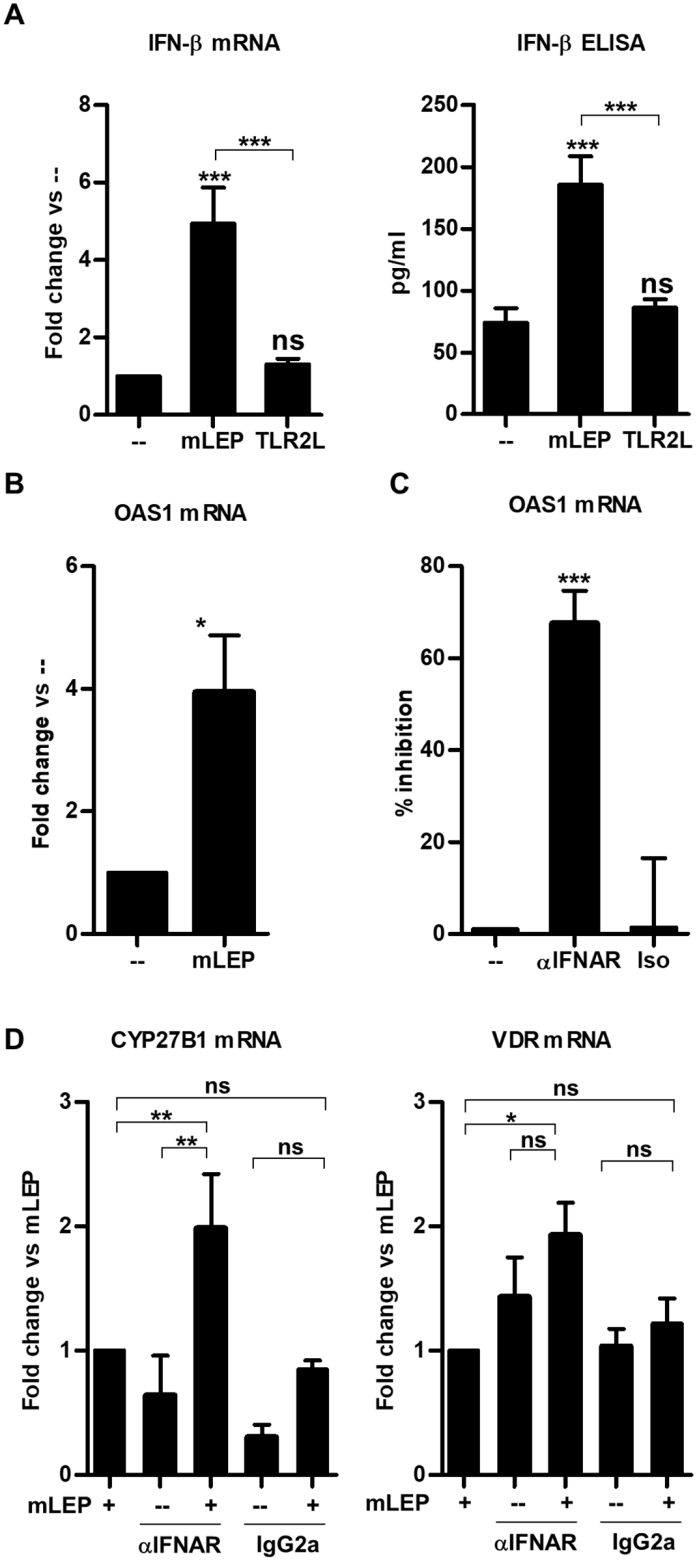
Blocking type I IFN signal during *M*. *leprae* infection relieves repression of intrinsic induction of CYP27B1. (**A**) Primary human monocytes were infected with live *M*. *leprae* at MOI of 10 for 6 hours for mRNA and 24 hours for culture supernatants. Interferon beta 1 (IFN-β) expression was measured using quantitative PCR and protein levels measured using ELISA [17]. qPCR data are shown as mean fold change vs. media ± SEM, n = 5, and ELISA are shown as mean concentration ± SEM, n = 5. (**B**) Primary human monocytes were infected with *M*. *leprae* at MOI of 10 for 24 hours, and OAS1 gene expression was measured by qPCR. Data are shown as mean fold change vs. media ± SEM, n = 4. (**C**) Primary human monocytes were pre-treated with a monoclonal neutralizing antibody against the type I IFN receptor (IFNAR) or isotype control then infected with *M*. *leprae* for 24 hours. OAS1 gene expression was determined by qPCR and represented as percent inhibition. Data are shown as mean ± SEM, n = 8. (**D**) Monocytes were pretreated with IFNAR neutralizing antibody or isotype control then infected with *M*. *leprae* at MOI of 10 for 24 hours, then CYP27B1 and VDR gene expression levels were measured using quantitative PCR. Data are shown as mean ± SEM, n = 8. Statistical significance was determined using one-way ANOVA. (*p ≤ 0.05, **p ≤ 0.01, ***p ≤ 0.001).

Given our previous studies demonstrating that *M*. *leprae* induces monocytes to release a broad array of cytokines [14, 17], we hypothesized that induction of specific cytokines downregulates CYP27B1 expression. Of the known cytokines triggered in monocytes and MΦ during *M*. *leprae* infection, IFN-β [17] has been shown to downregulate host immune responses [17]. In particular, IFN-β inhibits IFN-γ induced CYP27B1 expression in *M*. *leprae* infected cells [20, 21]. We therefore hypothesized that *M*. *leprae* induction of type I IFN inhibits the intrinsic upregulation of CYP27B1 in the infected cells. Mining unpublished data from our previous study [17], *M*. *leprae* infection significantly induced mRNA expression of IFN-β and IFN-β protein; however, TLR2L had no effect (Fig 2A). In new experiments, *M*. *leprae*-infected monocytes also significantly expressed type I IFN downstream gene product, OAS1 by qPCR, indicating the presence of a type I IFN response (Fig 2B). Addition of a neutralizing monoclonal antibody against the type I IFN receptor (IFNAR) to the monocyte cultures prior to *M*. *leprae* infection inhibited OAS1 expression (Fig 2C), confirming activation of the type I IFN pathway during *M*. *leprae* infection. In the same experiments, neutralization of IFNAR during *M*. *leprae* infection resulted in a significant induction of CYP27B1 and VDR gene expression (Fig 2D), suggesting that *M*. *leprae* induction of type I IFN blocks activation of an intrinsic response leading to upregulation of CYP27B1.

**Fig 3:**
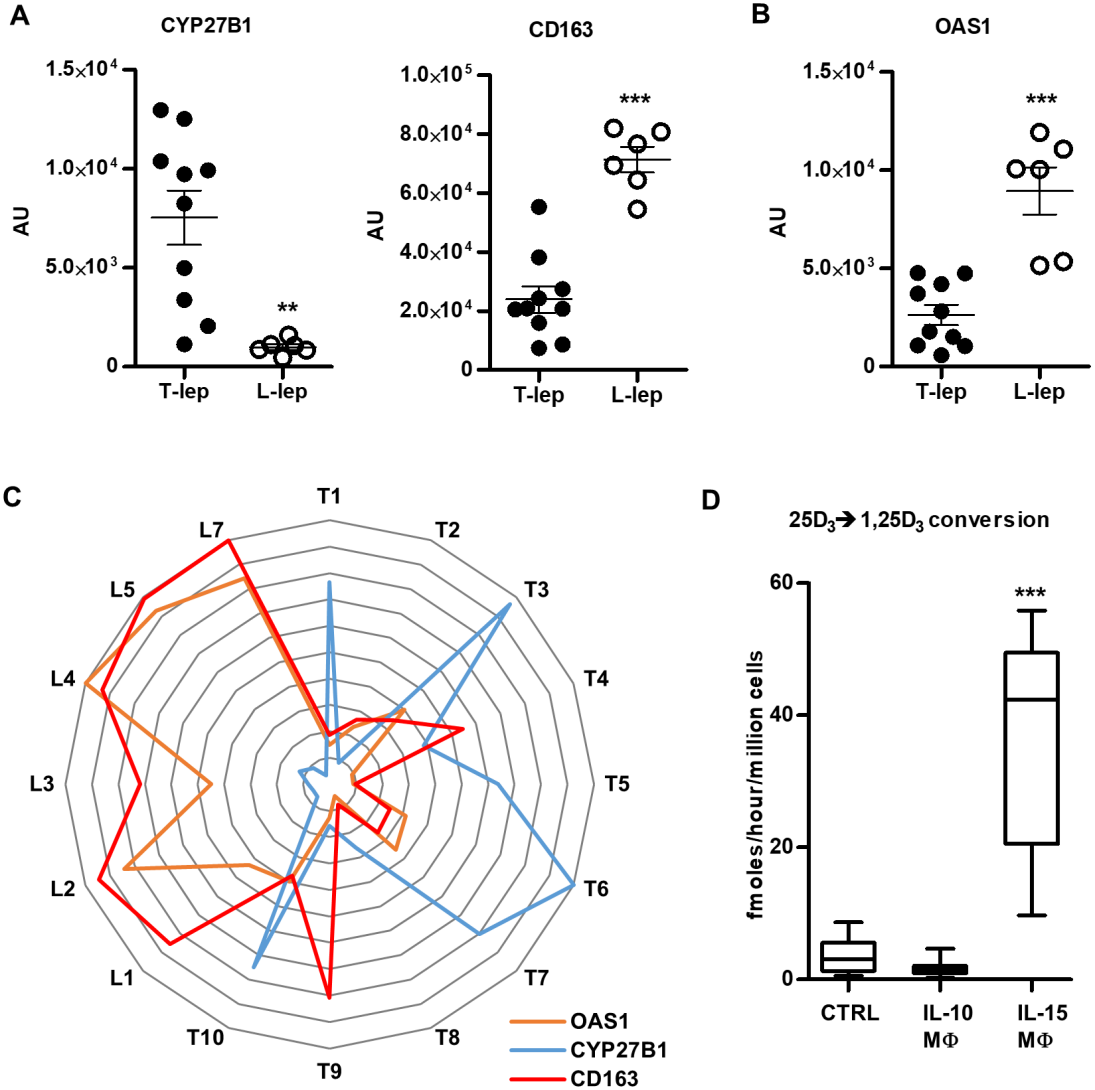
CYP27B1 gene expression is negatively correlated with OAS1 gene expression in *in vitro* studies and in leprosy lesions. (A) Correlation of CYP27B1 and CD163 as well as (B) OAS1 gene expression from microarray analysis of T-lep (n = 6) and L-lep (n = 5) human skin lesions. (C) Radar plot of CYP27B1, CD163 and OAS1 expression in T-lep and L-lep lesions normalized to the highest value in the experiment. (D) Primary human monocytes were treated with 10μg/mL IL-10 or 200ng/mL IL-15 for 48 hours. Control (Ctrl) MΦ were derived by treating monocytes with 10^3^ U/mL IL-4 for 48 hours. IL-10 MΦ and IL-15 MΦ were incubated with radiolabeled 25D_3_ for 5 hours and conversion of 25D_3_ to 1,25D_3_ was measured by HPLC. Data are shown as mean ± SEM, n = 3. Statistical significance was determined using one-way ANOVA. (**p ≤ 0.01, ***p ≤ 0.001).

Monocytes are initially recruited to the site of infection where they encounter microbial pathogens, but over time develop into tissue MΦ that contribute to outcome of the infection. In leprosy, T-lep lesions are characterized by M1-like MΦ and a vitamin D antimicrobial gene program; whereas, in L-lep lesions, M2-like MΦ predominate. To further explore the relationship between MΦ, the vitamin D antimicrobial and type I IFN pathways, we interrogated our previously published microarray study [17]. CYP27B1 expression was significantly greater in T-lep vs. L-lep lesions, and in 9/10 T-lep lesions greater than any of the six L-lep lesions (Fig 3A). Conversely, CD163, a marker of the M2-like MΦ in L-lep lesions, was significantly greater in L-lep vs. T-lep lesions, with the expression in 5/6 L-lep lesions greater than any of the T-lep lesions (Fig 3A). Given that IFN-β, a type I IFN, protein and mRNA are differentially expressed in L-lep vs. T-lep lesions, and impacts MΦ function [17], we examined mRNA levels for OAS1, which was significantly higher in L-lep lesions (Fig 3B). Linear regression analysis indicates that CD163 and OAS1 expression are coordinately expressed (P = 0.0004, R_2_ = 0.6019) and two way ANOVA analysis confirms that leprosy type of the lesion is significantly (P = 0.0003) associated with differential gene expression of CYP27B1, CD163 and OAS1 (Fig 3C).

**Fig 4:**
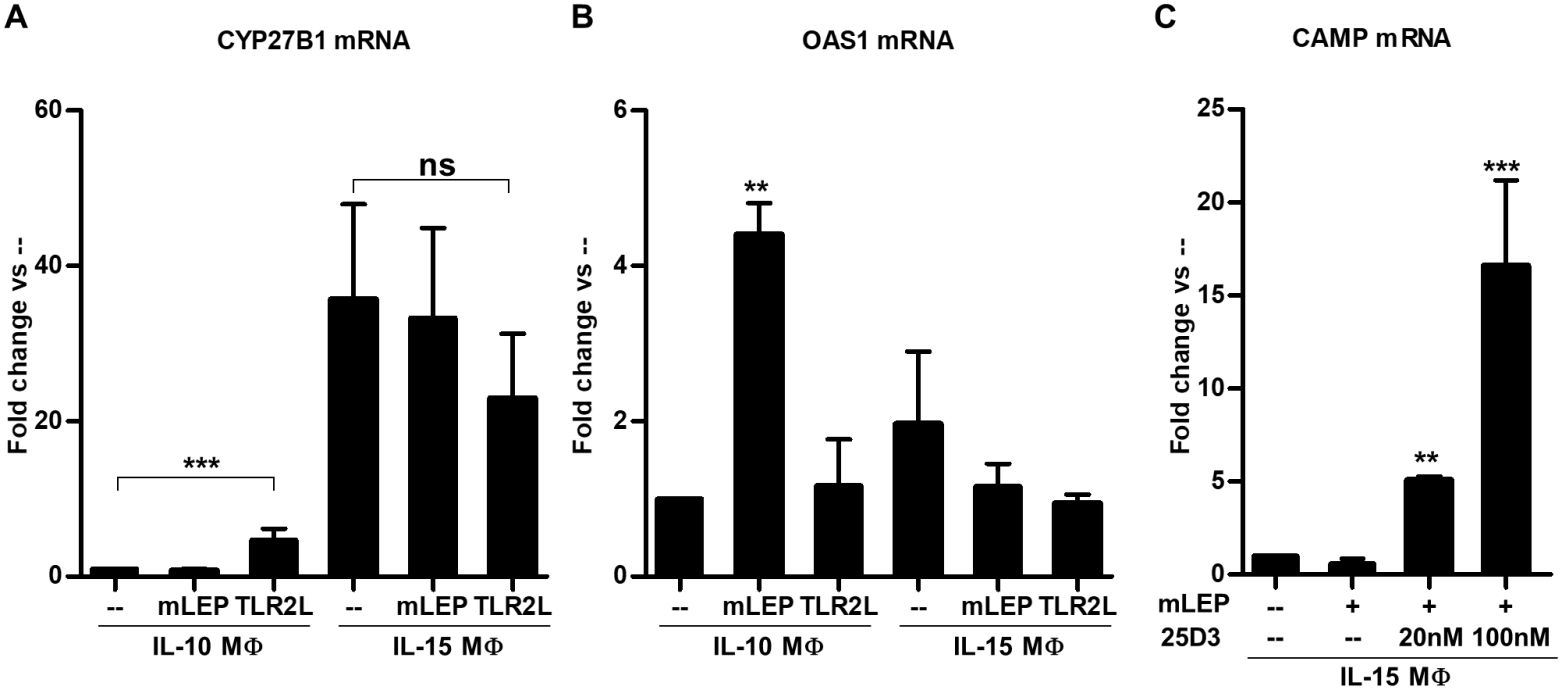
IL-15 MΦ and IL-10 MΦ differentially respond to *M*. *leprae* infection. IL-15 MΦ and IL-10 MΦ were infected with *M*. *leprae* or stimulated with TLR2L for 24 hours. (**A**) CYP27B1 and (**B**) OAS1 gene expression levels were assayed by quantitative PCR. (**C**) IL-15 MΦ were infected with *M*. *leprae* and supplemented with 20nM 25D_3_, 100nM 25D_3_, or vehicle control for 24 hours. Cathelicidin (CAMP) gene expression was assayed by qPCR. Data are shown as mean fold change ± SEM, n = 3. Statistical significance was determined using one-way ANOVA. (*p ≤ 0.05, **p ≤ 0.01, ***p ≤ 0.001).

To model the MΦ subtypes in leprosy lesions, we studied MΦ subsets derived *in vitro* with IL-15- vs. IL-10-treated monocytes yielding M1-like vs. M2-like MΦ, respectively. We previously described the IL-15- vs. IL-10-derived MΦ, to be phenotypically similar and functionally consistent with MΦ subtypes found in leprosy lesions [5, 13, 22]. We differentiated MΦ by treating monocytes with IL-10 or IL-15 for 48 hours, then determined their ability to metabolize vitamin D using HPLC as we have described above. Consistent with previous findings [5], IL-15 MΦ, but not IL-10 MΦ converted 25D_3_ to 1,25D_3_ (Fig 3D), which correlates with our *in situ* microarray and protein studies. To ascertain the effects of *M*. *leprae* infection on the vitamin D metabolic system in MΦ, we infected IL-10 MΦ and IL-15 MΦ with *M*. *leprae*, and measured CYP27B1and OAS1 mRNA expression by qPCR. *M*. *leprae* infection of IL-10 MΦ did not alter CYP27B1 mRNA expression, although exogenous TLR2 stimulation induced a significant upregulation (Fig 4A). On the other hand, CYP27B1 gene expression in IL-15 MΦ, which have high CYP27B1 gene expression at baseline, was not significantly affected as a result of either infection or stimulation with exogenous TLR2L (Fig 4A). In the same experiments, *M*. *leprae* infection of IL-10 MΦ led to the significant induction of OAS1 gene expression, but not stimulation with exogenous TLR2L (Fig 4B). In contrast, neither *M*. *leprae* infection not TLR2/1 activation of IL-15 MΦ induced upregulation OAS1 as a result of infection or following TLR2/1 stimulation. These findings suggest that in distinct MΦ subsets differentially regulate the vitamin D and type I IFN pathways.

To determine whether the vitamin D antimicrobial pathway was functional in *M*. *leprae* infected IL-15 MΦ, we added 25D_3_ to infected cells and measured the vitamin D-dependent antimicrobial peptide, cathelicidin (CAMP). Indeed, when exogenous 25D_3_ was added to *M*. *leprae* infected IL-15 MΦ at various doses, CAMP gene expression was induced in a dose-dependent fashion (Fig 4C), suggesting that these M1-like MΦ retain the intrinsic ability to engage the vitamin D-dependent antimicrobial pathway during *M*. *leprae* infection in the presence of sufficient 25D_3_ prohormone.

**Fig 5:**
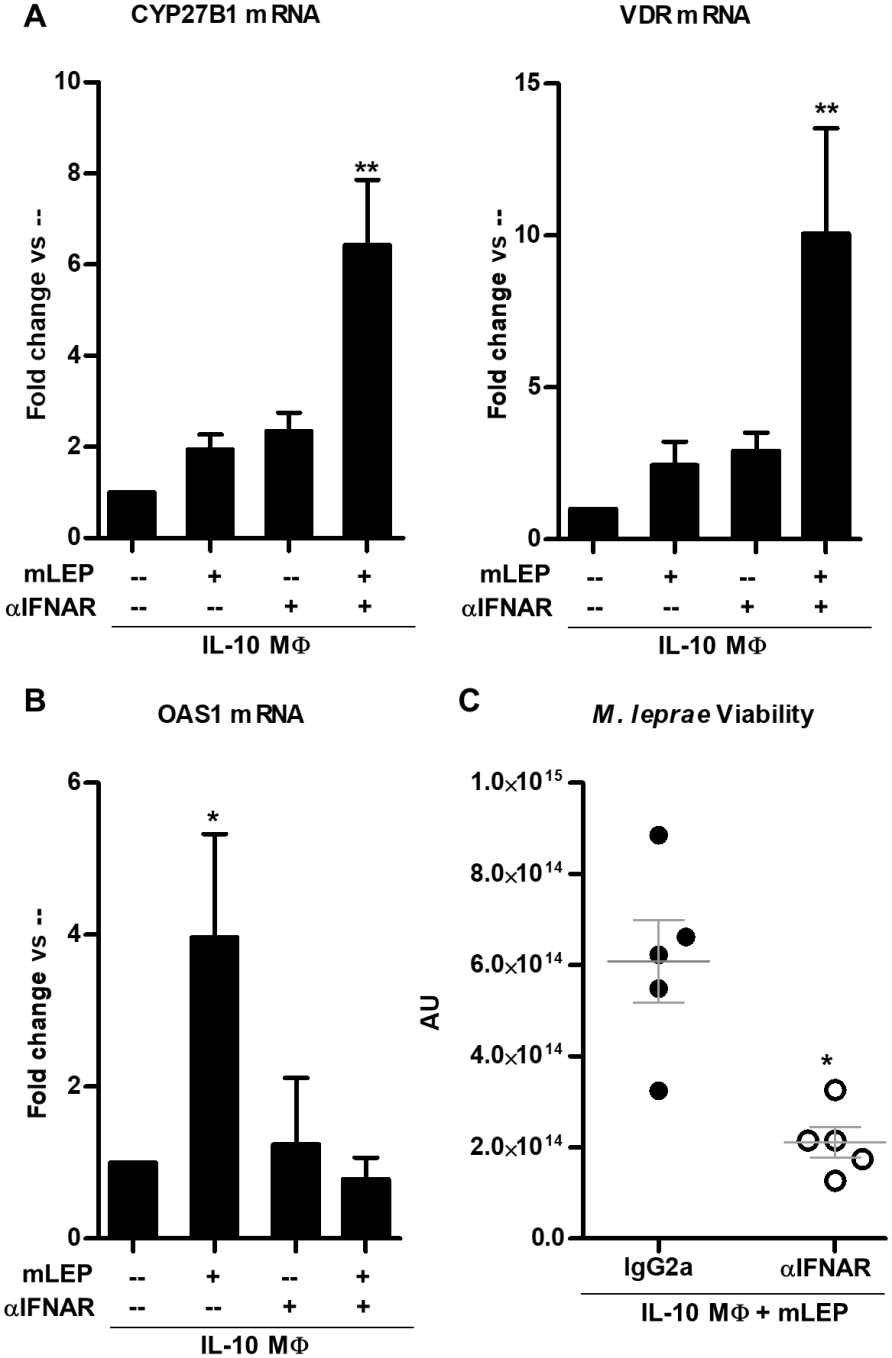
Blocking type I IFN signaling during *M*. *leprae* infection of IL-10 MΦ reduces bacterial viability. IL-10 MΦ were pre-treated with either antibody against the type I IFN receptor, IFNAR, or isotype control then infected with *M*. *leprae* at an MOI of 10 for 24 hours. (**A**) CYP27B1, VDR, and (**B**) OAS1 gene expression levels were measured using qPCR. Data are shown as mean ± SEM, n < 5. (**C**) IL-10 MΦ were infected with *M*. *leprae* and co-treated with IFNAR neutralizing monoclonal antibody, or isotype control for 5 days under vitamin D-sufficient conditions. The ratio of *M*. *leprae* 16S RNA to RLEP DNA was calculated as a measurement of bacterial viability. Statistical significance was determined using one-way ANOVA. (*p ≤ 0.05, **p ≤ 0.01).

The induction of the type I IFN response by *M*. *leprae* in infected IL-10 MΦ but not IL-15 MΦ, led us to explore the functional consequences of this cytokine response on the intrinsic induction of the vitamin D antimicrobial pathway. IL-10 MΦ were treated with a neutralizing monoclonal antibody specific for IFNAR and then infected with live *M*. *leprae*. The addition of neutralizing IFNAR antibody restored an intrinsic activation pathway leading to upregulation of CYP27B1 and VDR mRNA (Fig 5A), but conversely, inhibited expression of the type I IFN downstream gene OAS1 (Fig 5B). These data indicate that intrinsic activation of the vitamin D-dependent antimicrobial pathway during *M*. *leprae* infection is inhibited by the ability of the bacteria to induce type I IFN. Finally, we addressed whether the induction of type I IFN by *M*. *leprae* regulated an intrinsic antimicrobial response against *M*. *leprae*. In *M*. *leprae*-infected IL-10 MΦ cultured with 25D-sufficient human serum (44 ng/ml of 25D), treatment with the IFNAR neutralizing monoclonal antibody significantly reduced *M*. *leprae* viability as compared to the isotype control (Fig 5C). These results suggest that *M*. *leprae*, upon infection of M2-like MΦ, evades activation of the intrinsic antimicrobial response by invoking the type I IFN pathway. In summary, the regulation of the vitamin D antimicrobial pathway in M1-like vs. M2-like MΦ is distinct, and contributes to the outcome of the innate immune response to *M*. *leprae*.

## Discussion

Cells of the monocyte/MΦ lineage mount an innate antimicrobial response to defend against infection by an intracellular pathogen, yet the microbe has evolved to counter this intrinsic capacity to kill the foreign invader. Although the exogenous addition of purified ligands (TLR2L) derived from the pathogen provides an extrinsic mechanism to activate a vitamin D-dependent antimicrobial response in infected MΦ, the intrinsic activation of innate antimicrobial responses by the pathogen is not sustained [3]. Here we studied leprosy as a model to ascertain whether MΦ are capable of an intrinsic antimicrobial response to infection by *M*. *leprae*. We provide evidence here that infection of human MΦ by *M*. *leprae* intrinsically activates the vitamin D antimicrobial pathway as part of the innate immune response, but the bacterium blocks this response via the induction of type I IFN.

There is evidence for the existence of intrinsic anti-mycobacterial responses, although these are not usually sustained, and escaping the initial antimicrobial response is key for the pathogen to establish long term infection [3]. The identification of distinct MΦ gene expression profiles induced according to virulence of the infecting strain of *M*. *tuberculosis* suggests that subversion of the initial host response is critical to establishing infection [23]. Therefore, the ability of the invading pathogen to modulate the intrinsic macrophage response, such as antagonizing TLR2 [24] or suppressing the vitamin D-dependent antimicrobial response, will lead to immunopathology. Here, we demonstrate that monocytes and MΦ have the capacity to trigger an intrinsic antimicrobial response during *M*. *leprae* infection in the absence of exogenous triggers (TLR2L or IFN-γ), which is inhibited by the aberrant infection-induced expression of type I IFN. *M*. *leprae* may activate the intrinsic immune response in MΦ through a variety of pattern recognition receptors including TLR4, TLR9 and NOD2, but most importantly, TLR2 via the 19kDa [19], 33kDa [19, 25] and mLEP major membrane protein-II lipoproteins [26], since TLR2 activation leads to induction of CYP27B1 expression [2].

The intrinsic activation of the vitamin D antimicrobial pathway requires the metabolic conversion by the CYP27B1 enzyme of 25D, the inactive circulating prohormone (25D) and requires into the bioactive form (1,25D3) to transactivate its cognate receptor, the vitamin D receptor (VDR). Activation of human monocytes and MΦ by exogenous innate and adaptive immune signals (such as TLR2/1 and IFN-γ, respectively) have been shown to induce expression and function of CYP27B1 [1, 2]. Our data demonstrate that in contrast to activation with exogenously added TLR2 ligand or IFN-γ, monocytes infected with live *M*. *leprae* showed little intrinsic induction of CYP27B1 expression or enzyme activity. When the type I IFN receptor (IFNAR) was neutralized during *M*. *leprae* infection, the intrinsic induction of CYP27B1 during *M*. *leprae* infection was uncovered. This suppression of CYP27B1 has *in vivo* relevance, as we found that CYP27B1 is more highly expressed in T-lep vs. L-lep lesions, the self-limited vs. progressive forms of leprosy, respectively, and was inversely correlated with type I IFN signaling. Using our previously characterized and described *in vitro* models of the L-lep and T-lep resident MΦ (M2-like IL-10 MΦ and M1-like IL-15 MΦ) [5], we demonstrated that *M*. *leprae* infection of IL-10 MΦ induces a type I IFN response that inhibits CYP27B1 expression, similar to observations in monocytes. In contrast, IL-15 MΦ maintain their CYP27B1 expression and function in the presence of *M*. *leprae* infection. These data indicate that the regulation of CYP27B1 in infected MΦ at the site of disease is critical to activation of the intrinsic antimicrobial response.

The induction of type I IFN is a well-studied host defense mechanism against viral infection; however, their role in the immune response against intracellular infection with mycobacteria and bacteria is less defined [27]. While type I IFN are critical to clearance of viral infections, the same type I IFN response mediates suppression of antibacterial responses, leading to secondary bacterial infections, such as *Streptococcus pneumonia* [28]. The fact that the robust expression of type I IFN and downstream genes along with low CYP27B1 expression is characteristic of L-lep lesions and vice versa in T-lep lesions [17], suggests that the ability of type I IFN to inhibit CYP27B1 contributes to the outcome of the host response against mycobacteria in leprosy. Given that the neutralization of type I IFN uncovers the intrinsic induction of CYP27B1 expression following *M*. *leprae* infection *in vitro*, the induction of type I IFN provides an escape mechanism by which the bacterium subverts the vitamin D-mediated antibacterial response. The ability of type I IFN to inhibit CYP27B1 expression is likely related to the production of the immunosuppressive cytokine IL-10, which has been shown to be induced by type I IFN during *M*. *leprae* infection and inhibit CYP27B1 expression [14, 17]. Alternatively, the CYP27B1 promoter region contains an IRF8 (-543) response element as determined by MotifMap (http://motifmap.ics.uci.edu/), which is a type I IFN inducible transcription factor with known gene suppression functions [29]. Other studies have suggested additional mechanisms by which *M*. *leprae* blocks host defenses, some mediated through the mycobacterial cell wall component phenolic glycolipid 1 (PGL-1). Several studies have shown that PGL-1 manipulates host defense mechanisms such as complement activation, phagocytosis as well as cytokine release to inhibit maturation of dendritic cells and modulate T cell responses [30–33]; all of which enables survival of the bacteria. With regard to vitamin D-mediated antibacterial responses, our previous study showed that microRNA-21 (hsa-miR-21) was highly expressed in L-lep lesions vs. T-lep lesions and inhibited CYP27B1 gene expression and function [14]. Similar to the results presented here, hsa-miR-21 induction was exclusive to live *M*. *leprae* infection and not induced by purified TLR2 ligands [14]. Importantly, neutralization of the type I IFN pathway in *M*. *leprae* infected IL-10 MΦ resulted in decreased bacterial viability by uncovering the intrinsic vitamin D-mediated antimicrobial response. Indeed, further understanding of the pathways by which *M*. *leprae* initiates immune inhibitory mechanisms such as induction of the type I IFN pathway will provide novel therapeutic targets for mycobacterial diseases.

There is a well characterized genetic and functional association of the vitamin D pathway with leprosy. Single nucleotide polymorphisms in the VDR gene are associated with the different forms of leprosy [15, 34], as is expression levels of the protein itself [35]. However, the use of vitamin D to treat mycobacterial disease has been studied in clinical trials, which have shown inconsistent benefits [6, 10, 11, 36–39]. Our findings suggest a possible explanation for the varied outcomes. The efficacy of elevated systemic 25D levels in affecting local antimicrobial responses at the site of infection is predicated on the ability of the innate immune cells to convert the circulating 25D to 1,25D at the site of infection. Thus, if the pathogen bearing MΦ, such as those found in L-lep lesions, were unable to convert 25D, it would not be surprising to see minimal therapeutic benefit following vitamin D supplementation. More broadly, our findings suggest that the clinical management of mycobacterial disease using vitamin D supplementation will require simultaneous management of the vitamin D metabolic system to achieve therapeutic benefit. In conclusion, our results demonstrate that the intrinsic capacity of cells to activate antimicrobial defense mechanisms as part of the innate response, versus the ability of the pathogen to mask these responses, is a critical determinant of the outcome of infection.

## Supporting information

S1 Data Excel spreadsheet of raw data used to generate figures. (XLSX)
